# “What do they mean?” a systematic review on the interpretation, usage and acceptability of “they”

**DOI:** 10.3389/fpsyg.2024.1253356

**Published:** 2024-04-05

**Authors:** Mafalda Batista da Costa, Harriet R. Tenenbaum, Alexandra Grandison

**Affiliations:** School of Psychology, University of Surrey, Guildford, United Kingdom

**Keywords:** gender, language, pronouns, gender-neutral language, systematic review

## Abstract

The rise of feminist and LGBTQIA+ movements paved the way for many equality reforms. These include language reforms, which facilitate inclusion of multiple groups in society. For example, the shift from the generic “he” to “he or she” and “they” allows for the inclusion of women, transgender, and non-binary individuals in many narratives. For this reason, many institutions worldwide encourage neutral language. It remains unclear how individuals interpret neutral language. One case of neutral language is the pronoun “they,” which has been assigned multiple definitions from the 1970s to 2022. We examine how the pronoun “they” has been interpreted, used, and accepted over time. We discuss trends in the findings and make suggestions for future research directions, including the need for better methods to investigate pronouns and clarification on what the focus of neutral language should be. This timely commentary has implications for action on equality, diversity, and inclusion.

## Introduction

Language aids individuals in conceptualizing the world around them. Indeed, research demonstrates the impact of language on cognition and culture (for a review see Boroditsky, 2019). Given the close relationship between culture and language, Sniezek and Jazwinski (1986) suggest that the views of an androcentric society are demonstrated linguistically. Bodine (1975) presents this eloquently: “dictated by an androcentric world-view; linguistically, human beings were to be considered male unless proven otherwise” (p. 133), a statement that remains prevalent today (see Bailey et al., 2019). One way that language influences androcentrism is via personal pronouns, which are terms used to identify an individual. The current systematic review identifies how one solution to androcentrism in language, the pronoun “they,” has been interpreted historically, through a review of literature from as early as 1970 up until 2022. The review aims to inform subsequent research directions and future interventions to support equality, diversity, and inclusion.

Throughout history, there has been a shift in how pronouns, and in particular pronouns used generically, are understood. For example, the pronoun “he” has historically been used as a generic referent, as well as a masculine referent. The pronoun “they” has been interpreted in different ways throughout history. Changes in how pronouns are defined are related to legal and societal changes. For example, the rise of the LGBTQIA+ movement in the 2000s brought legal changes that allowed individuals to identify with their chosen gender. In Sweden, the pronoun “hen” was adopted as an entirely neutral pronoun (Gustafsson Sendén et al., 2015). In English speaking countries, a similar attempt was made with neopronouns (“ze” and “hir”) but has not progressed beyond the LGBTQIA+ communities (Crawford and Fox, 2007). A common alternative is the pronoun “they,” which is defined as a plural referent, a generic referent, a singular indefinite pronoun antecedent, and a referent for a single person who identifies as non-binary (Merriam-Webster, 2022). However, given how many definitions it holds, it is not clear how “they” is understood and interpreted today.

### The generic “he”

The concept of androcentrism was first coined by Gilman (1911), who wrote that society rests on the assumption that men are the ideal human type, whereas women are an accompaniment. In an androcentric society, men are seen as “the measure of all things” (Bailey et al., 2019). These beliefs manifest in language.

Others have argued against the androcentric worldview’s impact on language, stating that intention is enough to guarantee both clarity and equity (see Martyna, 1980). Martyna questioned whether “he” could be intentionally utilized in a purely generic form. Moreover, what is the guarantee that the user of “he” means it generically without explicitly stating so? Intent, however, in the usage of “he” is not the only problem. The recipient’s understanding of “he” is key too – utilizing “he” as a generic pronoun does not mean the recipient will understand it in the intended sense and may instead interpret “he” as denoting a man (Sniezek and Jazwinski, 1986). Many (e.g., Kidd, 1971; Soto, 1976; Moulton et al., 1978; MacKay and Fulkerson, 1979; MacKay, 1980; Switzer, 1990; Miller and James, 2009; Khan and Daneman, 2011) have shown that “he” evokes masculine imagery. What this implies is that “he” seems to function as a male-specific pronoun. Therefore, it is not that “he” cannot function generically but that “he” cannot function in a purely generic form.

Movements such as feminism, LGBTQIA+, and transgender communities have argued that a generic form needs to be introduced for equal language. This argument is often misunderstood as a radical language change that would eradicate specific and generic forms of “he,” which feminist linguists noted back in the late 1900s (Martyna, 1980). However, the movements do not aim to exclude men from the narrative. The opposition to the generic “he” is based on a desire for both equal rights and equal language – in other words, to create social equality that would allow for fair communication about genders (Martyna, 1980) and individuals who do not identify with a gender (Wayne, 2005).

### Is “he or she” the solution?

Because the pronoun “he” is not appropriate to be a generic referent, researchers have tended to utilize “he or she” as one of the pronoun options. The latter has been referred to as a feminization strategy that aims to produce more gender-neutral language. Research has suggested that “he or she” produces a degree of gender neutrality. For example, Hyde (1984) reported that “he or she” significantly produced the highest percentage of feminine stories (42%) compared to other pronouns (“he” and “they”). For “he or she” there was an almost equal balance of stories with masculine and feminine characters (58% of the characters in the stories for “he or she” were men). Gastil (1990) and Switzer (1990) showed similar findings, reporting that “he or she” produced a balance of feminine and masculine images. Lindqvist et al. (2019) supports such findings. In their study “he or she” did not evoke a masculine-stereotyped bias in a recruitment advert. Overall, what these findings suggest is that the combination of the pronouns “he or she” appear to function more generically than the pronoun “he.”

At the same time, the order of “he or she” is unbalanced in that “he” precedes “she.” Sexist prescriptions have existed in English to order men before women in binomial phrases to mark differences in people’s worth (Bodine, 1975). This concept dates back centuries as early philosophers claimed that linguistically, gender ordering should be according to the worthier of the pair being set first (Wilson, 1553, as cited in Bodine, 1975). Perhaps part of the reason “he or she” cannot be considered entirely gender-neutral may result from a masculine bias in research. Hegarty et al. (2016) note that research that focuses on male-first binomials identify these as gender-neutral or gender-fair language despite an influence of male dominance on order preferences. In sum, it seems that using “he or she” does not lead to inclusivity in language.

Research on the gendered binomial order demonstrates the ubiquity of a masculine bias. Kesebir (2017) explored the permanence of the “male-first pattern” in a variety of words across a range of English corpora, including “he or she.” Their results show that in texts published in the 20th Century, “he or she” is more likely to start with “he” instead of “she” (97.5%). The lack of variability (i.e., often described as “frozen order” by linguists) of “he or she” poses a problem because linguists have identified word order as relevant to social categories. For example, the more powerful element of a binomial phrase is more likely to be mentioned first (Benor and Levy, 2006). Thus, the “male-first pattern” may reflect a society where “men are the measure of all things” (Bailey et al., 2019). Nevertheless, preferences for male-first forms of several gendered binomials have weakened since the 1970s (Mollin, 2013), which coincides with the increasing support for gender equality and gender-fair language in many English-speaking countries (Hegarty et al., 2016). Nonetheless, the use of male-first linguistic forms serve to reinforce gender-stereotypes in a recurrent pattern. For example, Kesebir (2017) demonstrated that linguistic choice in gendered conjoined phrases such as “mother and father” and “businessman and businesswoman” matter because individuals assign more relevancy to the first mention rather than the second mention in a conjoined phrase. Thus, language such as “he or she” helps maintain androcentric worldviews, and as a result, maintains the oppressive social order that marginalizes non-men.

There is an additional concern with the generic “he” and “he or she”: neither “he” nor “he or she” can be genderless. With the growth of the transgender community, the gender binary of “male and female” is becoming unrepresentative because many individuals do not identify with their birth gender. Whilst much research has found that “he or she” has the potential to evoke gender-neutral imagery (e.g., Hyde, 1984; Gastil, 1990; Lindqvist et al., 2019), that imagery seems exclusive to women and men and not the genders who do not fit in the binary. In fact, in their investigation of which pronouns native English speakers use to refer to a genderless pronoun, LaScotte (2016) found that participants did not always envision someone outside of the gender binary when they used “he or she.” As a result, “he or she” reinforces the gender binary (Saguy and Williams, 2022), which researchers have suggested should be replaced with a multiple-category gender/sex system, of which the categories are not mutually exclusive (Hyde et al., 2019). To solve this problem, in terms of gender-fair language, we need to determine which alternative pronoun possesses the ability to evoke imagery beyond the gender binary.

### A genderless and gender-neutral pronoun

To identify an effective alternative to the problems mentioned in this review (essentially, the generic “he” and “he or she” as masculine-biased pronouns and thus, unrepresentative of women and those who identify beyond the gender binary), we need to establish an alternative that represents everyone. The representative would need to include individuals who identify with a gender and individuals who do not identify with any gender. Therefore, the pronoun is expected to be gender-neutral and genderless simultaneously.

In 2012, Sweden introduced a third person pronoun that is exclusively genderless/gender-neutral: “hen.” “Hen” is meant to be used generically when the gender is unknown or irrelevant, or to identify a transgender or genderless individual (Gustafsson Sendén et al., 2015). Sendén et al. explored the attitudes to the introduction of “hen” from 2012 to 2015, as well as the use of the Swedish third-person pronoun. Attitudes to “hen” have changed over time, beginning with negative attitudes that became more positive as of 2015. There was a concomitant change in behavior. In 2013 and 2014, participants reported to rarely using “hen.” By 2015 the use of “hen” increased by 25%. Left-wing orientation and low sexism predicted individuals’ use of “hen” (19% of participants). Conversely, higher levels of right-wing orientation and sexism was associated with lower use of “hen” (15% of participants). Attitudes to “hen” changed faster than behavior. Making grammatical changes to a language is a difficult process (Paterson, 2014). However, “hen” demonstrates that language can change. In fact, Gustafsson Sendén et al. (2021) reported that, from 2015 to 2018, individuals have continued to perceive “hen” more positively. The introduction of “hen” and the changes in attitudes with regards to the gender-neutral/genderless Swedish pronoun give way for other languages to introduce their own gender-neutral/genderless pronoun. A similar attempt has been made in English-speaking countries with less success. Indeed, transgender communities have introduced “ze” and “hir” as genderless pronouns. However, these pronouns have not been very widespread outside LGBTQIA+ and transgender communities (Crawford and Fox, 2007).

The grammatical structure in the English language as it exists facilitates the use of a gender-neutral/genderless pronoun because it already contains a pronoun that has been interpreted as gender-neutral by researchers and even linguists: “they” (Quirk et al., 1972, 1985; Wales, 1996). As a result, gender-fair language has been encouraged by a variety of institutions. For example, the American Psychological Association (2020) recommended that authors “do not use the generic ‘he’ or ‘he or she’ to refer to a generic person; instead, authors must rewrite the sentence or use the singular ‘they’. When writing about a known individual, use that person’s identified pronouns” (paragraph 29). Additionally, in 2008, the European Parliament released guidelines that encourage the use of gender-neutral language and advise members to avoid the use of the generic “he” and “man.” The United Nations in 2018 also released guidelines on gender-fair language, advising users to only make gender visible when it is relevant for communication as well as providing inclusive examples of language, e.g., “humankind.” In countries like Australia, for example, use of non-sexist language has been encouraged for several decades (e.g., the new national anthem was changed to become more inclusive, “Australia sons” became “Australians all”; Strahan, 2008). Similarly, the Canadian’s Department of Justice encourages the use of gender-neutral language (Department of Justice Canada, 2023). In non-English speaking countries, gender-fair language has been encouraged as well, e.g., the Netherlands and Germany (Weida, 2022). Furthermore, there are many countries whose language is already genderless, such as Armenian, Persian and Swahili. Many academic institutions worldwide encourage gender-fair language. The National Centre for State Courts has released guidance on how courts can encourage gender-inclusive language (Wirkus and Zarnow, 2023). In 2022, the Supreme Court of the Philippines approved guidelines on the use of gender-fair language in the Judiciary and Gender-Fair Courtroom Etiquette, and the Courts and Tribunals Judiciary in the United Kingdom released guidelines on gender equality in 2013, encouraging users to use gender-neutral language. It is worth noting that there are countries that oppose the inclusion of gender-fair language. For example, Argentina implemented a policy in June 2022 that forbade public educational institutions from using gender-neutral language on the basis of grammatical sanctity (Lankes, 2022). In France, the French Senate passed a bill banning inclusive language (Bollinger, 2023), claiming that their masculine form acts as a neutral form. Nevertheless, these examples suggest that a wide variety of institutions are becoming increasingly aware of the importance of fair and inclusive language and are encouraging their members to follow.

When individuals use “they,” it can have positive effects. “They” produces gender-neutral/inclusive imagery in comparison to “he” and, to some extent, “he or she.” For example, Switzer (1990) presented children with a brief scenario (in which the pronoun differed for each participant) before asking multiple questions about the character. “They” aided children in producing inclusive imagery. Additionally, Conkright et al. (2000) explored the effects of pronoun type on children’s recall and interpretation of stories. In their findings, the authors showed that the pronoun “they” was evenly interpreted as either generic or as a specific gender. LaScotte (2016) investigated which pronouns native English speakers used when referring to a genderless person (i.e., “the ideal student*”*). Most participants (79%) used a gender-inclusive approach to describe the target (i.e., using “he or she” or singular “they”), with 68% of the participants using singular “they.” What this may mean is that “they” as a generic pronoun may be useful to help individuals express less gendered ideals and help them create a more inclusive worldwide view. Indeed, utilizing the pronoun “they” as gender-neutral/genderless may be an alternative to the generic “he” and “he or she.”

Nevertheless, even if “they” appears to be the alternate pronoun to encourage genderless imagery and ideals, research has found mixed results with regard to the pronoun “they.” For example, some report that “they” produces a mix of gender-neutral imagery (Switzer, 1990; Conkright et al., 2000), whereas others report that “they” also works to produce gender specific imagery (Lindqvist et al., 2019). Martyna (1980) argued that although users intend to use the pronoun “he” generically, its use is not restricted to these uses. Similar issues arise with “they.” Bodine (1975) explained that grammarians identified the pronoun “they” as plural in the 19th Century, which means that users may use “they” as a plural referent, as well as a specific-gender singular referent, and not just a purely genderless pronoun.

Lindqvist et al. (2019) investigated the effect of three language strategies on the reduction of the masculine bias through a recruitment candidate advert. The language utilized in the advert varied according to the language strategy. In the paired strategy, the advert used “he or she.” In the traditional neutral strategy, the advert either used a gender-neutral noun (“the applicant”) or the singular gender-neutral “they.” Finally, in the new gender-neutral strategy, the non-gendered pronoun “ze” was used. Lindqvist et al. found that the wording used in the description of the candidate significantly impacted the participants’ gender associations. The gender-neutral noun “the applicant” and the singular gender-neutral pronoun “they” possessed a masculine bias. In other words, participants were more likely to choose a man for the candidate. When “he or she” or “ze” was utilized, there was no masculine bias. With regards to “ze,” familiarity with the pronoun did not significantly impact participants’ gender association of the candidate. Most participants were familiar with the singular “they” but were less familiar with the other pronouns, such as “ze.” Lindqvist et al. suggest that the singular “they” is not in the same category as the newly created gender-neutral alternatives. Instead, “they” is a familiar pronoun with a different use (i.e., singular rather than the conventional plural).

Combined, the evidence suggests two main conclusions. First, “he” does not function generically, and it is not a gender-neutral nor a genderless pronoun. Secondly, binomials (“he or she”) are a better alternative to the generic “he” as a gender-neutral pronoun but not interpreted as a genderless pronoun. As a result, this systematic review will not investigate either of these topics. We focus on understanding how people use, accept, and interpret the pronoun “they” over recent history. Thus, this review examines what “they” means, how individuals use the pronoun “they,” and the acceptance of the pronoun “they” over time. This review will examine literature from the 1970s to 2022. Based on Gustafsson Sendén et al.’s (2015) findings, which demonstrate a change over time regarding the Swedish pronoun “hen,” we hypothesize that “they” will become more gender-neutral, increase in use, and rise in acceptability over time.

## Method

### Search strategy

We searched two digital databases, PsychINFO and Web of Science, for empirical research that investigated the interpretation of the pronoun “they.” We chose PsychINFO because it contains information related to psychology, mental health, and the behavioral and social sciences. Web of Science allows the search to be conducted across a wide range of science, social science, and humanities databases simultaneously. In Supplementary Table S1 we show the initial search terms, their combinations, and the results from the searches.

After the initial search, we re-evaluated the terms and searched synonyms. Supplementary Table S2 displays these terms, their combinations, and the results.

Because the two main searches did not yield many results, we conducted a third search with terms taken from Ansara and Hegarty (2013). We display the results from the third search in Table 1.

**Table 1:** Final search terms, combinations, and results.

Database	Search term combinations		Number of papers emerged	Number of papers selected
PsychINFO	*Sexist language*	Pronoun*	15	2
	*Gender-Biased Language*	Pronoun*	6	1
	*Gender Bias in Language*	Pronoun*	5	2
	*Sex Biased Language*	Pronoun*	1	0
	*Sex Bias in Language*	Pronoun*	3	0
	*Linguistic Sexism*	Pronoun*	4	0
**Total**			34	5
Web of Science	*Sexist Language*	Pronoun*	47	3
	*Gender-Biased Language*	Pronoun*	25	0
	*Gender Bias in Language*	Pronoun*	89	0
	*Sex Biased Language*	Pronoun*	12	0
	*Sex Bias in Language*	Pronoun*	38	0
	*Linguistic Sexism*	Pronoun*	10	0
Total			221	3

**Figure 1:**
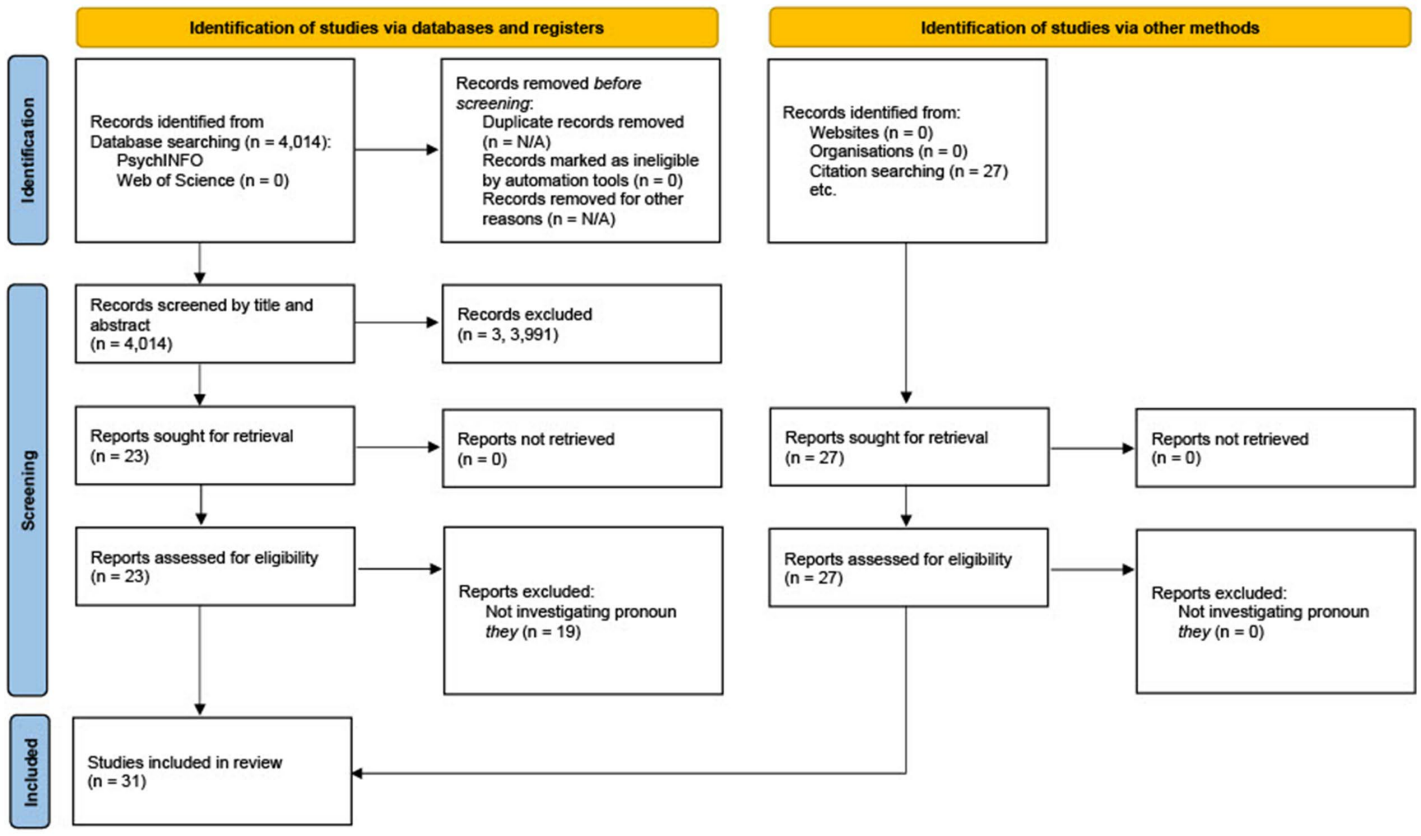
PRISMA flow diagram for search results (Page et al., 2021).

An additional digital database, Google Scholar, was used in the search for empirical research after the initial searches did not yield many results. However, this search did not result in new papers. We conducted manual searches from the reference lists of the included articles, as well as Google Scholar citation searches, which resulted in 28 papers. Studies were selected based on being published between 1970 and 2022, written in English, and investigating the pronoun “they” in the English language. A Zotero digital library was used to safekeep the studies selected. Studies were included if they included empirical research investigating participants’ interpretation of the pronoun “they” – that is, whether the pronoun “they” refers to individuals of the gender binary, beyond the gender binary or a combination. Studies that investigated other pronouns, including “they,” were included in the review. Figure 1 includes a PRISMA flow diagram of the process by which the papers were identified and then eliminated.

### Study sample

Table 2 displays a summary of the characteristics (e.g., the year of publication, the samples, the methodology of the empirical studies) in the review. Table 3 shows the relevant outcomes.

**Table 2:** A summary of the research articles’ samples and methods.

Study	Study population						
	Total	Sex/Gender	Age	Occupation	Location of data collection	Other	Study method
Martyna (1978)	40	20 females; 20 males		Stanford University students	United States of America		Complete sentences, orally and written; complete a questionnaire which asks participants to reflect on how they had decided which pronouns to use, and to describe an image or idea that came to mind of the character.
Moulton et al. (1978)	490	226 male; 264 female		College students	United States of America		Make up a story creating a fictional character who fits a student theme. Thirty sentences with gaps to be filled.
Hyde (1984)	310	140 boys/men; 170 girls/women	5–21	60 first graders; 67 third graders; 59 fifth graders; 121 college students	United States of America		Make-up a story about a character; asked participants if character was a boy or a girl.
	132	64 boys; 68 girls	8–12	59 third graders; 73 fifth graders	United States of America		
Fisk (1985)	72	36 boys; 36 girls		36 kindergartens; 36 first graders	United States of America	Middle-class, public elementary school.	Listened to a story and were asked to retell it. Shown a picture of a young boy or young girl and were asked to identify who the story was about.
Khosroshahi (1989)	55	28 women; 27 men		Harvard University students in Psychology courses	United States of America		Presented with written paragraphs and were asked to sketch their mental imagery in response to it. Were asked whether they could give the person they drew a name or age.
					Kroepelin (1989)			60	30 males; 30 females	17–30	12 Freshmen; 14 Sophomores; 17 Juniors; 14 Seniors; 3 Graduate Students	United States of America		Write a paragraph about a fictional character according to the pronoun allocated.
Gastil (1990)	93	48 women; 45 men		Midwestern University	United States of America		Verbally describe image that comes to mind when reading 12 sentences. Participants were asked if the images that came to mind were male, female, mixed or neither.
Switzer (1990)	471	233 females; 238 males		225 first graders; 246 seventh graders	United States of America		Heard beginning of a story and were asked to write a brief ending to the story, as well as name the student in the story.
Prentice (1994)	109	50 females; 59 males		Students	United States of America		Had language corrected on lab reports for 12 weeks. Students completed Cognitive Tasks Questionnaires – free association task (complete sentences), a story generation task (given 10 themes and asked to write a short description of the story and a name for the main character).
Bennett-Kastor (1996)	36	19 males; 17 females	9–12	Fourth, fifth, and sixth grade students in Wichita, Kansas, public schools.	United States of America		Completed survey comprehension task, where participants were asked whether the sentence was about a male, a female or either one. Asked whether different occupations were “mostly male,” “mostly female” or “either male or female.”
	27	18 females; 9 males	9–12		United States of America		Write a story on the day in the life of a masculine, feminine or neutral referent.
Prögler-Rössler (1997)	121		14–15; 18–19	Fifth grade pupils of a Nottingham Comprehensive School; First year Science students at Nottingham Trent University	United Kingdom		Survey to elicit spontaneous pronoun usage as well as explanations of their own usage. Test of separate sentences with gaps to be filled, as well as asked to reflect back on own pronoun usage in the questionnaire.
Conkright et al. (2000)	48	24 girls; 24 boys	24 6-year-olds; 24 9-year-olds		United States of America		Short passages were presented; free recall, cued recall and gender assignment.
Lee (2007)	269	121 women; 148 men	17–21	102 High School Students; 167 University Students	Hong Kong	All Cantonese speakers who studied English as a second language for 7 years or more.	Four types of tests: a translation test, a tag test, a slot-filling test, and a proofreading test.
Strahan (2008)	17				Australia	17 first year essays on Child Language Acquisition	Language analysis on essays.
Garnham et al. (2012)	36	5 men; 31 women	22.03 (mean age)	University of Sussex students	United Kingdom		Read passage, presented one sentence at a time and had to decide for each sentence pair whether the end was a sensible continuation of the first sentence.
LaScotte (2016)	38	24 females; 14 males	18–59		United States of America	All native English speakers.	Verbal response to questions regarding “ideal student.”
Doherty and Conklin (2017)	38		Second year undergraduates		United Kingdom	Native English speakers.	Naturalness rating task.
Noll et al. (2018)	98			Psychology Undergraduate Students	United States of America	All native English speakers.	Agree/disagree with sentences.
	49			Psychology Undergraduate Students	United States of America	All native English speakers.
Bradley et al. (2019)	123	77 women; 41 men; 4 non-binary; 1 agender	18–101		United States of America	English speaking participants	Read descriptions of scholarship applications, and rated students academically. Also selected from an array of photos which person they thought was the one they read about.
Lindqvist et al. (2019)	411	145 women; 252 men; 5 transwomen; 4 transmen, 3 non-binary; 2 unknown	35.8 (mean age)		Sweden and United States of America		Read about a candidate applying for a job as real estate agent and were asked which one of four photos they associated with the candidate.
Ahokas (2020)	34	19 female; 15 male	20–33		Sweden and Finland	All studied English for at least 10 years.	Interview (where asked to describe the ideal student) and questionnaire (with two sections: modify sentences and report attitudes toward gender-neutral language and issues).
Bradley (2020)	222	142 women, 69 men, 11 nonbinary, 9 transgender.	18–63		United States of America, Canada, United Kingdom	Native English speakers.	Sentence judgments, Prescriptivism Inventory and Ambivalent Sexism Inventory.
Hekanaho (2020)	1,128	411 cis-female; 611 cis-male, 101 transgender (79 non-binary)	Under 40		Finland and Sweden	Non-native and native English speakers.	Survey study, focusing on three aspects: usage, acceptability and attitudes.
Konnelly and Cowper (2020)	8 speakers	5 non-binary,3 binary-gendered			Not stated		Judgments of innovative variety of English, in which singular they can be used to refer to definite, singular individuals of any gender and can take antecedents.
		Moulton et al. (2020)						40				Not stated	Native English speakers.	Sentence acceptability judgment task.
Stormbom (2020)	40 journals; 1,003 articles				Bulgaria, Canada, Finland, India, Indonesia, Italy, Japan, Netherlands, Norway, Pakistan, Poland, Singapore, South Africa, South Korean, Spain, Sweden, Switzerland, Turkey, United Kingdom and United States of America	The Corpus of Open Access Journal Articles	Examination of use of epicene pronouns in Open Access journals.
von der Malsburg et al. (2020)	24,863				United States of America		Belief estimation, text completion and self-paced reading tasks.
Arnold et al. (2021)	150				United Status, Australia, and the United Kingdom		To read two-sentence stories and answer two questions.
LaScotte (2021)	34	16 female; 18 male	18–40	Intensive English Program students.	United States of America	Non-native English speakers	Verbal response to questions regarding “ideal student”.
Yakut et al. (2021)	100 papers				Not stated	Corpus-based approach.	Explore epicene pronoun usage in English when academic writers in Social Sciences refer to gender-unknown singular human referents in publications.
Zhang and Yang (2021)	1,248,476 words in 4950 text samples			University students	China	Corpus of Written English Corpus of Chinese Learners.	Corpus study to investigate how English learners in China use epicene pronouns.
Saguy and Williams (2022)	54	35 non-cisgender; 18 *cis* women; 1 *cis* man	18–76	28 LGBTQ+ organizations; 8 feministic organizations; 15 educators in gender and sexuality.	United States of America	Progressive gender activists.	Interviews, which focused on expertise and activism; asked to reflect on the term gender neutral generally and as it is used in association with specific issues. Ask questions on gender-neutral pronouns, idea of eliminating gender-specific pronouns and using gender-neutral pronouns for everyone.

**Table 3:** A summary of the research articles’ relevant outcomes and ratings for the quality assessment of each research article.

Study	Relevant outcomes
Martyna (1978)	“… however, we found ‘they’ appearing more often in spoken responses…” (p. 134)
	“This may simply be because ‘they’ is easier to say than ‘he or she’, and we are more used to singular ‘they’ in our colloquial speech than the ‘he or she’ construction. It may also reflect our reluctance to use ‘they’ for a singular subject in writing, as we have been taught that it is grammatically incorrect.” (p. 135)
Moulton et al. (1978)	“We determined the gender of the fictional characters from pronouns and proper names used in the story and from a follow-up question that asked their subjects to name their fictional characters if they had not already done so. (…) for ‘their’, 46% were female (...).” (p. 1034)
Hyde (1984)	“When the pronoun was ‘he’ or ‘his’, overall 12% of the stories were about females; when it was ‘they’ or ‘their’, 18% were female, and when the pronoun was ‘his’ or ‘her’ (‘he’ or ‘she’), 42% of the stories were about females.” (p. 700)
	“There was a significant effect of pronoun on sex of story character, (…) with 17% female stories when the pronoun was ‘he’, 31% for ‘they’, 18% for ‘he or she’, and 77% for ‘she’.” (p. 702)
	“However, when the truly neutral pronoun ‘they’” is used, the percentage of female stories is still substantially below 50% (18% in Experiment 1, 31% in Experiment 2).” (p. 705)
Fisk (1985)	“For each group as a whole, clearly the ‘they’ and ‘s/he’ presentations functioned in a non-male-biased manner, with 68% and 77% of choices not being male.” (p. 484)
Khosroshahi (1989)	“‘He or she’ and ‘they’ did not differ significantly in the number of female, (...) male, (...) and generic images (...) they elicited in the minds of the students. Similarly, ‘he’ and ‘they’ did not differ in the number of female, (...) male, (...) and generic figures, (...) they evoked.” (p. 515)
	“The data suggest that from the perspective of a feminist, ‘he or she’ is best, ‘he’ is worst, and ‘they’ in between. Thus overall, ‘he or she’ evoked the highest number of female images (34%), ‘he’ the lowest number (19%), and ‘they’ an intermediate number (26%).” (p. 516)
Kroepelin (1989)	“When given ‘their’, 65% wrote about a male and 35% wrote about a female.” (p. 92)
	“Did the writing of male or female depend on the given of ‘his’, ‘his/her’, and ‘their’? No, it could not be statistically determined that the writing of male or female depended on the given of ‘his’, ‘his or her’, and ‘their’.” (p. 95)
Gastil (1990)	“Overall pronoun effects were highly significant, ‘he’ evoked more male images (…) than either ‘he/she’ (…) or ‘they’ (…).” (p. 635)
	“Regarding overall pronoun effects, ‘he’ brought to mind fewer mixed images (…) than either ‘he/she’ (…) or ‘they’ (…).” (p. 637)
	“Comparing overall pronoun effects, ‘they’ elicited more self-images (…) than ‘he’ (…).” (p. 638)
Switzer (1990)	“When subjects hear the ‘they’ story, the imagery they developed as 44.2% male, 27% female and 28.8% inclusive.” (pp. 77 and 79)
	“… use of the term ‘they’ generated more inclusive referents (28.7% compared to 7.9% for ‘he/she’).” (p. 79)
Prentice (1994)	“…female students generated less male imagery than male students, especially if they were exposed to reformed language.” (p. 12)
Bennett-Kastor (1996)	“A third of the test sentences contained the third-person plural form *their/themselves* with a singular antecedent, and 30.5% of these were interpreted as referring to “either a male or a female” (i.e., gender neutral readings) …” (p. 292)
	“*They* with a singular antecedent NP was associated most often with gender-neutral interpretations.” (p. 297)
	“Otherwise, *they* is the pronoun most likely to be interpreted neutrally, suggesting that, for these children, the unmarked gender of third person plural pronouns overrides number agreement.” (p. 298)
	“Rather, *they* was the most common anaphoric choice-used equally to refer to stereotypical males, females, or referents of either gender.” (p. 298)
Prögler-Rössler (1997)	“Figure 1 shows the pronoun usage in the test without distinguishing the individual noun groups. Pupils and students, males and females taken together, ‘they’ reached a score of 59.7% …” (p.37)
	“Even though this confirms the hypothesis that ‘he’ is used most often with stereotypically male nouns, this figure is still surprisingly low compared to the use of ‘they’ in the same noun group (43%).” (p. 38–39)
	“With the pronoun ‘they’ sex-specific imagery occurred rarely (5–7%), most people (54%) stated that they had not been thinking of anything in particular. The comparison of the pronoun ‘they’ used in singular, generic contexts with double pronoun constructions shows that the latter are more suitable to make women visible in language. (...) Even though double pronoun constructions seem to favor self-imagery, ‘they’ obviously is the preferred alternative. The reason for this preference can be seen in the answer of one female pupil who stated that she used *they* because it is “nice to be remembered,” others probably wanted to avoid the ‘clumsiness’ of double pronoun constructions.” (p. 42)
	“Thus it is also possible that the informants used ‘they’ more often than they thought they did or than they wanted to admit. The fact that this alternative is still not widely accepted by grammarians may have played an important role here.” (p. 43)
Conkright et al. (2000)	“… however, the third prediction was supported in that neither ‘they’ nor the alternating pronoun produced gender differences in recall.” (p. 491)
	“For girls, in the ‘they’/feminine and ‘they’/neutral conditions, gender assignment of “either” was based on the pronoun ‘they’ (rather than on activity) more often than in other gender/activity categories.” (p. 493)
	“… ‘they’ for girls appears to be a more generic choice of pronoun than it is for boys because girls’ gender understanding depended less on activity.” (p. 493)
Strahan (2008)	“‘They’ is very clearly being used with a specific referent in mind, whose gender is known not only to the writer but also to the reader.” (p. 26)
	“This pattern appears in the writing of many students, and I suggest that it is typical of Australian English, i.e., the pronoun ‘they’ is not just a third person plural and third person singular ‘indefinite gender’ or ‘general’ pronoun, but it is a third person ‘gender not relevant to the discussion’ pronoun. In discussion with several of these students, a recurring phrase was that the gender ‘just does not matter’.” (p. 27)
Lee (2007)	“Another noteworthy finding of the present study is that generic ‘they’ is not an uncommon usage among young Hong Kong people, especially when the context suggests a strong plural meaning, (…) All of the interviewees commented than when the context suggests plural meanings, they would tend to opt for the pronoun ‘they’. (...)” (pp. 291–292)
Garnham et al. (2012)	“In line with our expectations in English, the proportions of positive judgments and the positive judgment times revealed that the gender representation was biased by stereotyped information (or lack of it, in the case of the neutral items, so that they readily maps onto the representation of, say, “singers,” and both “men” and “women” in the second sentence are seen as equally consistent with that representation), as in Gygax et al. (2008).” (p. 497)
LaScotte (2016)	“The findings show that in the free response question the majority of participants use singular ‘they’ when referring to the singular, genderless antecedent “the ideal student.” Singular ‘they’ represents 55% of the 136 pronouns used to refer to this singular, genderless antecedent in response to this survey question.” (p. 67)
Doherty and Conklin (2017)	“They rated ‘them’ less natural than gender-matching pronouns but more natural than gender-mismatching pronouns.” (p. 723)
	“Comparisons of the naturalness ratings of *them* for each of the three antecedents (gender-known, high-expectancy and low-expectancy) revealed that participants rated ‘them’ with a low-expectancy antecedent as significantly more natural than with a high-expectancy one (...) and with a gender-known antecedent (...). Results also revealed that participants rated them more natural with a high-expectancy than with a gender-known antecedent (...).” (p.723)
Noll et al. (2018)	“(...) It may be that, at the time this study was conducted, participants were more familiar with ‘he’ being used as an epicene. In contrast, ‘they’ was a less familiar epicene that possible had a feminist connotation.” (p. 7)
	“(…) Both sets of results indicate that using ‘they’ as an epicene pronoun is more inclusive than is ‘he’ in that the activation of feminine words is affected by which pronoun is used.” (p. 10)
Bradley et al. (2019)	“Results indicate ‘they’ is a viable option for a gender-neutral and non-binary pronoun in English, contrary to previous results (…). ‘They’ appeared to be gender-neutral, which could represent a difference between the younger and perhaps more progressive sample of participants in our experiment compared to previous studies.” (p. 4)
	“Even those who do not know someone who goes by ‘they’ interpret it as gender-neutral, suggesting it may be a “naturally occurring” option for gender-neutrality and non-binariness.” (p. 4)
Lindqvist et al. (2019)	“The same result was obtained for the gender-neutral (singular) ‘they’, where 68.4% of the participants associated ‘they’ with a masculine gender, …” (p. 113)
Ahokas (2020)	“When looking at the written pronouns of the two groups combined (…). The second most common pronoun was *they* (20.6%).” (p. 11)
	“As can be seen, whereas ‘he/she’ was by far the most preferred choice in writing, ‘they’ was the most common in speech.” (p. 13)
	“None of the Finnish participants recalled being taught about the inclusive use of pronouns and the issues related to gendered ones. (…) Thus, learners resort to the combination ‘he/she’ because they are not taught about the possibility of using *they* as a reference to singular entities. (...) Both respondents were aware of singular ‘they’ but said that using it is sometimes confusing because they associate it with plural referents.” (p.18)
	“Firstly, ‘they’ was the most commonly occurring pronoun in speech for both groups. This is somewhat unexpected, especially for the Finns, because they preferred the ‘he/she’ construction in writing. One explanation for this is that speech is considered to be less formal than writing.” (p.19)
	“Even though it is argued that ‘they’ diminishes feminine gender because it still elicits more mental images of men than women (…) it does not mean that we should stop using it. (…) The reason for ‘they’ or ‘he/she’ evoking disproportionate number of male images does not mean that it is the word itself that evokes them but the culture behind it.” (p.20)
Bradley (2020)	“The patterns of results indicates that grammatical judgments of gender-neutral and non-binary uses of singular ‘they’ are related to both linguistic and non-linguistic psychological factors, and that these psychological factors include attitudes related to language and gender.” (p. 8)
	“These results confirm that prescriptivist attitudes do contribute to the rejection of certain uses of singular ‘they’. Crucially, prescriptivism was not the sole predictor of grammatical judgments: despite correlations between measures of linguistic and social attitudes, both prescriptivism and benevolent sexism independently predict negative judgments of specific types of gender-neutral uses of singular ‘they’, indicating that the causes of these judgments are likely heterogenous.” (p. 8)
Hekanaho (2020)	“Singular ‘they’ was both the most commonly used (about 80%) and most commonly accepted (94%) generic pronoun.” (p. 503)
	“Singular ‘they’ on the other hand was most commonly lauded for being gender inclusive.” (p. 504)
	“As regards nonbinary pronouns, the results demonstrate clearly that ‘they’ was acceptable to more participants (67%) than the neopronouns (33%). Overall, it seems it is easier to accept a familiar pronoun being used in a new context, then to accept completely new pronouns.” (p. 505)
	“Nearly all transgender participants (97%) accepted nonbinary pronouns (…). The cisgender participants were more divided, as cis men opposed nonbinary pronouns the most, 39% rejecting ‘they’ and 80% rejecting the neopronouns. In contrast, 73% of cis females participants accepted ‘they’, and 54% accepted the neopronouns.” (p. 505)
Konnelly and Cowper (2020)	“While some speakers find the use of singular ‘they’ in the innovative Stage 3 contexts described here to be objectionable for social reasons, these objections are all too frequently packaged as an effort to defend the grammar itself, or, in the case of linguists who take this position, as deriving inexorably from the grammar. Singular ‘they’ – and non-binary singular ‘they’ more specifically – provides an apt example of how grammar and social meaning are not so neatly separated.” (p.16)
Moulton et al. (2020)	“However, our results show that ‘they’-sentences were just as highly acceptable as singular gendered pronoun sentences regardless of whether the gender of the referent was known or unknown. Moreover, ratings for ‘they’-known condition were consistently high across participants.” (p. 6)
	“We found that the naturalness rating of referential singular ‘they’ with a gender known referent is less acceptable when used deictically in comparison to when used anaphorically with a gender-neutral antecedent.” (p. 9)
	“These results taken together lend support to the claim that non-innovative speakers find singular *they* less acceptable than a singular gendered pronoun. But this effect only arises reliably when the pronoun I used deictically.” (p. 9)
Stormbom (2020)	“The results of the corpus study suggest that the use of epicene pronouns is very much in a state of flux in present-day OA international publishing in English: overall, singular ‘they’ was the most commonly used pronoun (46.8%), followed by ‘he or she’ forms (38.5%) and generic ‘he’ (14.7%).” (p. 201)
von der Malsburg et al. (2020)	“… whereas expectations that the next president would be female largely manifested as ‘they’ references,…” (p. 126)
Arnold et al. (2021)	“Our critical finding is that explicitly introducing Alex’s pronouns promotes the singular interpretation of ‘they’.” (p. 1694)
LaScotte (2021)	“This demonstrates that regardless of proficiency level, international students use a range of pronouns to refer to singular, non-gender specified antecedent – even singular ‘they,’ which is not typically taught.” (p. 92)
Yakut et al. (2021)	“In this study, we focused on linguistic sexism in the pronoun system in several social science academic journals. With some fluctuations, the usage of *they* as a nonbinary singular pronoun has moved from a small percentage (…) to a noticeably larger percentage (...) across the years examined in this research; our observations also showed that the usages of *he* (...) and *she* (...) have maintained their dominance throughout 2010–2019 compared to ‘they’.” (p.8)
Zhang and Yang (2021)	“The result shows that the most popular epicene pronoun is generic ‘he’ followed by combination ‘he or she.’ While singular ‘they’ account for 15.0% of the total epicene pronoun. Generic ‘he’ is used quadruple the amount of singular ‘they’ in the corpus.” (p.7)
Saguy and Williams (2022)	“… initially responded to our open-ended question about “gender-neutral pronouns” by commenting specifically on the use of nonbinary personal pronouns. In contrast, very few responded to this opening question by discussing singular ‘they’ used either as a universal or as an indefinite pronoun...” (p. 18)
	“… raised the issue of using singular ‘they’ as a universal pronoun.” (p. 19)
	“… with several people recommending that people use ‘they/them’ pronouns when referring to someone whose self-identified pronouns are unknown.” (p. 21)
	“Several activists employed the term default to describe the practice of using singular ‘they’ until informed of a person’s self-identified pronouns.” (p. 21)
	“In the interviews, activists discussed three distinct usages of singular ‘they’: (1) as a nonbinary personal pronoun; (2) as a universal gender-neutral pronoun; and (3) as an indefinite pronoun.” (p. 22)

### Information extraction

Information such as year of publication, study samples, and methodology were extracted if included in the research paper. We extracted and then thematically analyzed findings relating to the pronoun “they.” Three categories emerged: meaning of “they,” use of “they,” and acceptance of “they.”

### Search results

In total, the database search revealed 4,014 publications, of which 23 were initially selected. However, of these 23 papers, 19 were excluded. The exclusion criterion for the search was papers that were not investigating the pronoun “they” and its interpretation. Duplicate papers were merged in the original database search and not retrieved for consideration. Overall, 4,010 publications were removed entirely. Further manual searches were conducted from the reference lists of the included articles, as well as Google Scholar citation searches. Twenty-eight papers emerged from these searches, resulting in 32 papers in total reviewed by two independent reviewers (see Figure 1 for PRISMA Flow Chart).

### Quality check

The 32 articles identified in the search results were assessed using the Quantitative Assessment Tool by Kmet et al. (2004). The tool’s criteria contains 14 items (see Table 4), which are scored on the basis of the extent to which the criteria was met: “Yes,” “Partial,” “No” and “Not Applicable.” For each research paper, a summary score was calculated using the assessment tool guidelines (see Table 5). Two reviewers independently performed quality assessments, and disagreements were solved by consensus. A score of 75% or over indicated strong quality, a score between 55 and 75% indicated moderate quality and a score of 55% or less indicated weak quality (from Van Cutsem et al., 2017).

**Table 4:** Criteria for assessing the quality of quantitative studies and overall results (from Kmet et al., 2004).

Criteria		Yes	Partial	No	N/A
1	Question/objective sufficiently described?	29	2	1	
2	Study design evidence and appropriate?	32			
3	Method of subject/comparison group selection or source of information/input variables described and appropriate?	30	1	1	
4	Subject (and comparison group, if applicable) characteristics sufficiently described?	27	3	1	1
5	If interventional and random allocation was possible, was it described?	5	4	1	22
6	If interventional and blinding of investigators was possible, was it reported?				32
7	If interventional and blinding of subjects was possible, was it reported?	11	1	1	19
8	Outcome and (if applicable) exposure measure(s) well defined and robust to measurement/misclassification bias? Means of assessment reported?	29	1		2
9	Sample size appropriate?	23	5	2	2
10	Analytic methods described/justified and appropriate?	26	4		2
11	Some estimate of variance is reported for the main results?	22	6	2	2
12	Controlled for confounding?	24	1		7
13	Results reported in sufficient detail?	31	1		
14	Conclusion supported by the results?	32			

**Table 5:** Quality assessment of research articles included in Systematic Review (from Kmet et al., 2004).

	Yes	Partial	No	N/A	Rating
Martyna (1978)	10	2		2	Strong
Moulton et al. (1978)	11	2		1	Strong
Hyde (1984)	11	1		2	Strong
Fisk (1985)	11	1	1	1	Strong
Khosroshahi (1989)	11	1		2	Strong
Kroepelin (1989)	10	1		3	Strong
Gastil (1990)	11			3	Strong
Switzer (1990)	13			1	Strong
Prentice (1994)	12			2	Strong
Bennett-Kastor (1996)	9	2	1	3	Strong
Prögler-Rössler (1997)	10	1		3	Strong
Conkright et al. (2000)	12			2	Strong
Strahan (2008)	6			8	Strong
Lee (2007)	12			2	Strong
Garnham et al. (2012)	11			3	Strong
LaScotte (2016)	12			2	Strong
Doherty and Conklin (2017)	8	1	4	1	Moderate
Noll et al. (2018)	11	1		2	Strong
Bradley et al. (2019)	9	2	1	2	Strong
Lindqvist et al. (2019)	12			2	Strong
Ahokas (2020)	10	1	1	2	Strong
Bradley (2020)	11			3	Strong
Hekanaho (2020)	6	3	1	4	Moderate
Konnelly and Cowper (2020)	5			9	Strong
Moulton et al. (2020)	11	1		2	Strong
Stormbom (2020)	10			4	Strong
von der Malsburg et al. (2020)	10	1		3	Strong
Arnold et al. (2021)	10	3		1	Strong
LaScotte (2021)	10	2		2	Strong
Yakut et al. (2021)	10			4	Strong
Zhang and Yang (2021)	8	2		4	Strong
Saguy and Williams (2022)	9	1		4	Strong

N/A indicates not applicable. Quality scores: >75% strong, 55–75% moderate, <55% weak.

## Results and discussion

### Overview

This study examined how the pronoun “they” is interpreted. We hypothesized that there would be a change of interpretation, use, and acceptability of “they” over time in which “they” would become more inclusive over the years. Through the examination of papers and analysis, we examined the meaning of “they,” the use of “they,” and the acceptance of “they.”

Figure 1 illustrates how we selected the studies. Thirty-two studies were included after full-text review: 21 on the meaning of “they,” nine on the use of “they” and six on the acceptance of “they.” Year of publication ranged from 1978 to 2022, with two studies conducted in the 1970s, three in the 1980s, five in the 1990s, three in the 2000s, six in the 2010s, and 13 studies in the 2020s. Out of the 32 studies, five had a child sample (3 to 12 years of age) and the remaining 27 were conducted with an adult sample. Twenty-one of the studies took place in the United States, whilst five were set in the United Kingdom, two in Australia, Finland, and Sweden, and one each in China, Hong Kong, and Canada. Two studies were corpus based, one of which extracted papers from multiple countries worldwide (see Table 4). One of the studies did not report which country they took place in. We present a summary of the studies’ year of publications, samples, method, and relevant outcomes in Tables 2, 3.

### The meaning of “they”

Because this review investigates the interpretation of the pronoun “they” across time, the results are presented in chronological order to present a timeline of how the pronoun “they” has changed over the years. Two early studies (Moulton et al., 1978; Hyde, 1984) report the pronoun “they” to be more masculine-biased, with fewer than 50% of participants referring to women in their story-creation tasks. Thus, early work suggests that “they” was masculine biased initially. This masculine bias seems to change in the mid-1980s. In 1985, the pronoun “they” was reported to function in a non-masculine biased manner, with 68% of choices in the character identification task being feminine (Fisk, 1985). This finding continues through 1989 to 1997, in which studies report that the pronoun “they” generates more inclusive imagery (Khosroshahi, 1989; Kroepelin, 1989; Gastil, 1990; Switzer, 1990; Prentice, 1994; Bennett-Kastor, 1996; Prögler-Rössler, 1997).

From the year 2000 onwards, we see a variety of results. For example, girls appear to use the pronoun “they” more generically than boys do (Conkright et al., 2000), which may be an effect of a continued prevalent androcentric worldview (see Bailey et al., 2019) where a masculine bias is still observed in language. Thus, girls use the pronoun “they” more generically than boys because it represents them too. The pronoun “they” also has a plural meaning as documented by Lee (2007). “They” also seems to have a “gender not relevant” meaning (Strahan, 2008, p. 28). This means that if the gender of the referent is not relevant or necessary to the conversation, the pronoun “they” was used. The pronoun “they” is a more inclusive pronoun than the pronoun “he” because the findings show more feminine connotations than “he” (Noll et al., 2018), and even acts as a gender-neutral pronoun (LaScotte, 2016), which is seen in 2019 as well (Bradley et al., 2019). However, in 2019, the pronoun “they” continues to possess a masculine bias (Lindqvist et al., 2019). In a study in 2020, “they” has a feminine association (von der Malsburg et al., 2020). In fact, data demonstrates that individuals use a range of pronouns (i.e., “he,” “she,” “they,” or “he or she”) to refer to a singular, non-gender antecedent (LaScotte, 2021).

In the 2020s, “they” is also used to refer to individuals who are non-binary who personally use the pronouns “they/them” (Arnold et al., 2021). Thus, in the 2020s, there are different interpretations of the singular pronoun “they.” Indeed, “they” is used as a non-binary personal pronoun, as a universal gender-neutral pronoun, and as an indefinite pronoun (Saguy and Williams, 2022).

Overall, there appears to be a linear progression in interpretation. In sum, the pronoun “they” was interpreted with a masculine bias in the 1980s and this linguistically reflected the views of an androcentric society (Sniezek and Jazwinski, 1986), as “human beings were to be considered male unless proven otherwise” (Bodine, 1975, p. 133). However, the second wave of feminism between the 1960s and 1970s (Pauwels, 2003) drew attention to gender biases in language as women felt excluded because of specific linguistic choices such as the use of the pronoun “he” as a generic pronoun (i.e., the pronoun “he” could refer to men and women). This shift in Western society may explain the move from the pronoun “they” possessing a masculine bias in the late 1970s and early 1980s to becoming more inclusive in the 1990s. However, this definition of inclusivity is restricted to the gender binary. It seems that in some of the aforementioned studies (e.g., Moulton et al., 1978; Hyde, 1984; Fisk, 1985; Khosroshahi, 1989; Kroepelin, 1989; Gastil, 1990; Switzer, 1990; Prentice, 1994), “they” possessing feminine connotations suggested it was an inclusive pronoun, but this does not represent genders other than women and men.

**Figure 2:**
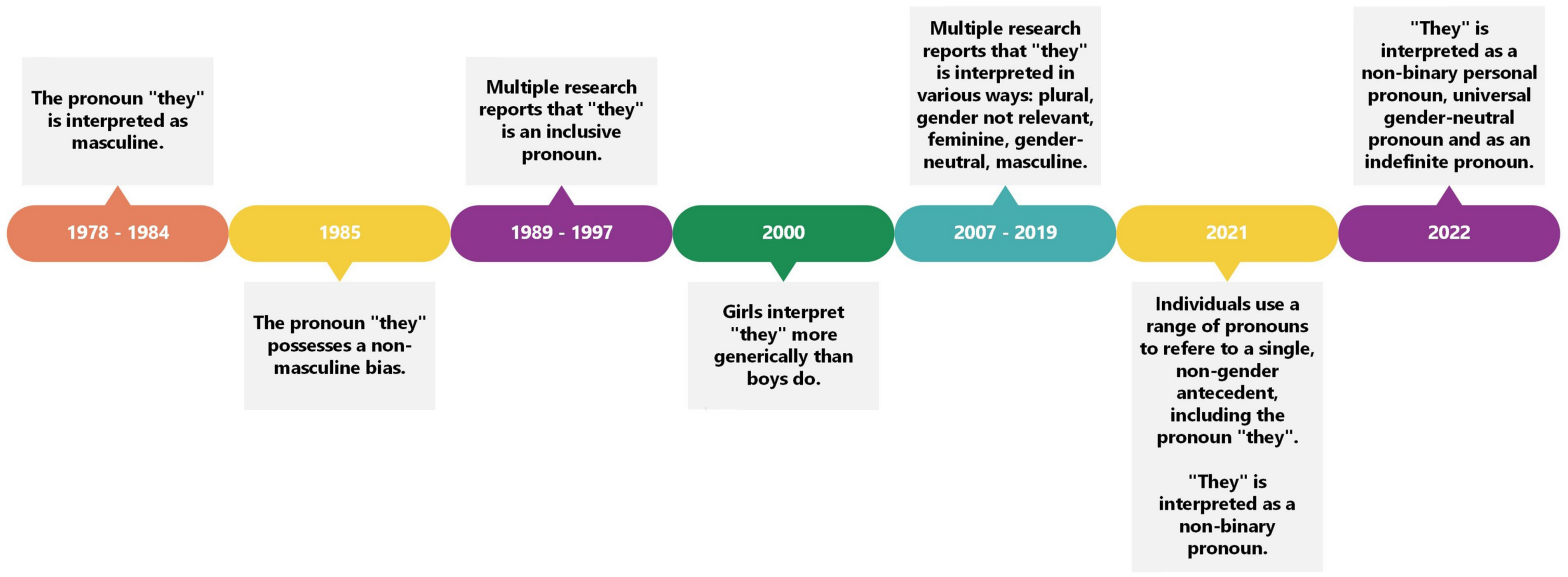
Timeline to show the meaning of the pronoun “they” through the years.

With the rise of the LGBTQIA+ movement in the 2000s, we witness the pronoun “they” shifting into a more neutral and genderless territory. The movement has impacted many legislative efforts to recognize gender identity all over the world. For example, the introduction of the 2004 Gender Recognition Act in the United Kingdom, which allows transgender people to identify with their chosen gender fully and legally and acquire a new birth certificate. In Canada, there was an adjustment of the Canadian Human Rights Act and the Criminal Code in 2017. Australia has passed multiple legislations since 2014 to recognize transgender rights, whilst Sweden has had legislations in place since 1972 and continues to re-evaluate their laws up until the current day. China and Hong Kong, on the other hand, have laws in place that recognize gender identity, however, only after post-sexual reassignment surgery. Similarly, in Finland, a legal gender reassignment on official documents is only recognized if the person is sterilized or for other reasons infertile. In the United States, as of February 2021, the Equality Act, which prohibits discrimination on the basis of sex, sexual orientation, and gender identity, was passed in the House of Representatives, and is awaiting consideration in the Senate. Laws regarding transgender rights differ from state to state, with many states in 2022 passing anti-LGBTQIA+ bills (Lavietes and Ramos, 2022). Nevertheless, many institutions such as the European Parliament (2008), the United Nations (2018), and the American Psychological Association (2020) encourage non-sexist and non-gendered language in their rules in recognition of fluid gender identity. Simultaneous with increased legal protection, a shift in interpretation is observed up until recent years, where the data reflects more fluid interpretations of the pronoun “they.” For example, “they” has masculine and feminine associations, as well as gender-neutral and non-binary interpretations. Although transgender rights differ worldwide, changes have occurred; research suggests that these changes are reflected in the pronoun “they” (Figure 2).

### The use of “they” in spoken and written English

If the pronoun “they” is interpreted as more generic through time, then it is expected to become more common in spoken and written English language. The studies examined in this systematic review investigated whether the pronoun “they” was used in speech or written language, how commonly it was used in comparison to other pronouns, and how the pronoun “they” can be used (as gender-neutral, masculine, feminine or non-binary). Martyna (1978) was the first to report findings on the use of the pronoun “they.” The pronoun “they” was more commonly used in spoken responses than in written responses possibly because “they” is easier to say than “he or she” and individuals are more used to “they” in colloquial language. In the late 1990s, Bennett-Kastor (1996) reported children frequently chose the pronoun “they” (13.1%) more when writing a story than the pronouns “he” (11.9%) and “she” (5.6%). In addition, Prögler-Rössler (1997) recorded a written usage of 59% of the pronoun “they” by their student population compared to “s/he” (7%), “she” (9%), “he” (12%) and other (13%). More recently, there have been more reports on the usage of the pronoun “they.” For example, in written responses, the pronoun “they” was used 20.6% of the time (Ahokas, 2020) and in speech, the pronoun “they” was the most common choice. Hekanaho (2020) found singular “they” was the most commonly used (80%) pronoun in written responses. Stormbom (2020) conducted a search of gendered language in open research articles and reported that singular “they” was the most commonly used pronoun (46.8%). Similarly, Yakut et al. (2021) found that the usage of the pronoun “they” as a non-binary singular pronoun considerably increased from the year 2010 to the year 2019 in Social Science academic journals. In Chinese University students’ English writing, Zhang and Yang (2021) found that singular “they” accounted for 15% of the total pronoun usage in comparison to gendered pronouns.

**Figure 3:**
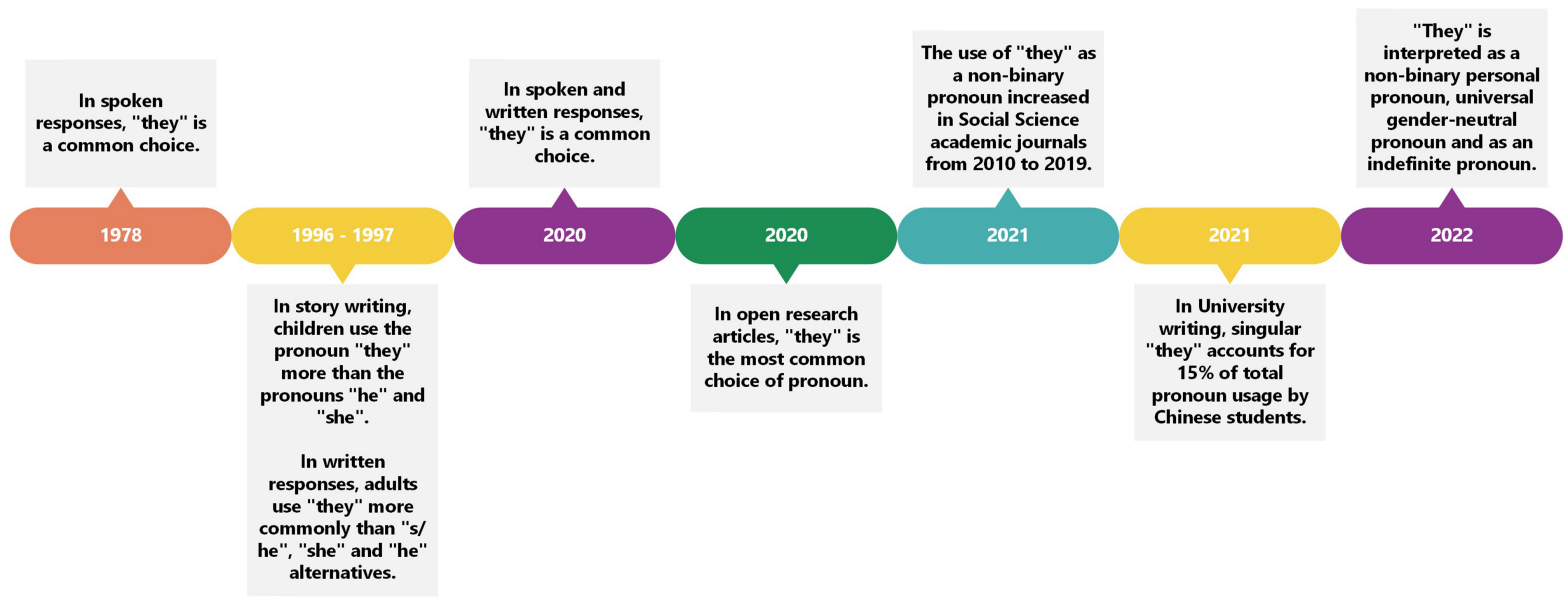
Timeline to show the use of the pronoun “they” through the years.

Overall, with time, the pronoun “they” became a common choice of pronoun, even in countries where the participants’ first language was not English (e.g., Ahokas, 2020; Hekanaho, 2020; Zhang and Yang, 2021). This increase in usage fits with historical context. With the rise of the LGBTQIA+ movement, and in particular, individuals in the movement who do not identify with their assigned sex or any specific gender (i.e., non-binary individuals, transgender individuals, and others), linguistic choices that refer to identity are changing. For example, non-binary people may adopt the pronoun “they” as their referent of choice. The history of the interpretation of the pronoun “they” suggests it has become more fluid and its meaning open to interpretation. For example, early research (Quirk et al., 1972, 1985; Wales, 1996) reported that the pronoun “they” was already interpreted and used as gender-neutral by researchers and even linguists. Moreover, more institutions are encouraging the use of non-sexist language. For example, the change in the lyrics of the Australian National anthem previously outlined, demonstrates a move away from masculine oriented language (“Australia sons”) to more gender neutral and inclusive language (“Australians all”; Strahan, 2008) (Figure 3).

### The acceptance of “they”

Given the increased familiarity of the pronoun “they” over time, we next turn to an examination of the acceptance of the pronoun “they.” More specifically, research has examined whether this pronoun has been accepted as a generic pronoun or a plural pronoun, and whether language structure impacts the rates of acceptance. Garnham et al. (2012) discovered that the pronoun “they” received positive judgment (i.e., participants approved of the sentence construction) when constructed with a plural word (“singers”) and binary genders (“men” and “women”). Doherty and Conklin (2017) reported that their participants rated the pronoun “they” as less natural than pronouns that matched the gender of the referent in the task but more natural than pronouns that did not match the gender of the referent shown. Konnelly and Cowper (2020) found participants objected to the use of singular “they” for social reasons – in particular, to defend the sanctity of grammar. Similarly, Bradley (2020) found that prescriptivist attitudes contributed to the rejection of certain uses of singular “they.” Moulton et al. (2020) reported that their participants thought sentences with “they” were just as acceptable as sentences with singular gendered pronouns (i.e., participants rated sentences for naturalness on a seven-point scale), despite whether the gender of the referent was known or unknown. Hekanaho (2020) reported that singular “they” was the most commonly accepted generic pronoun (94%), as well as the most accepted non-binary pronoun (67%). When looking at gender differences, 97% of transgender participants accepted non-binary pronouns. Meanwhile, 61% of cisgender male participants accepted “they” as a non-binary pronoun and 73% of cisgender female participants accepted “they” as a non-binary pronoun.

**Figure 4:**
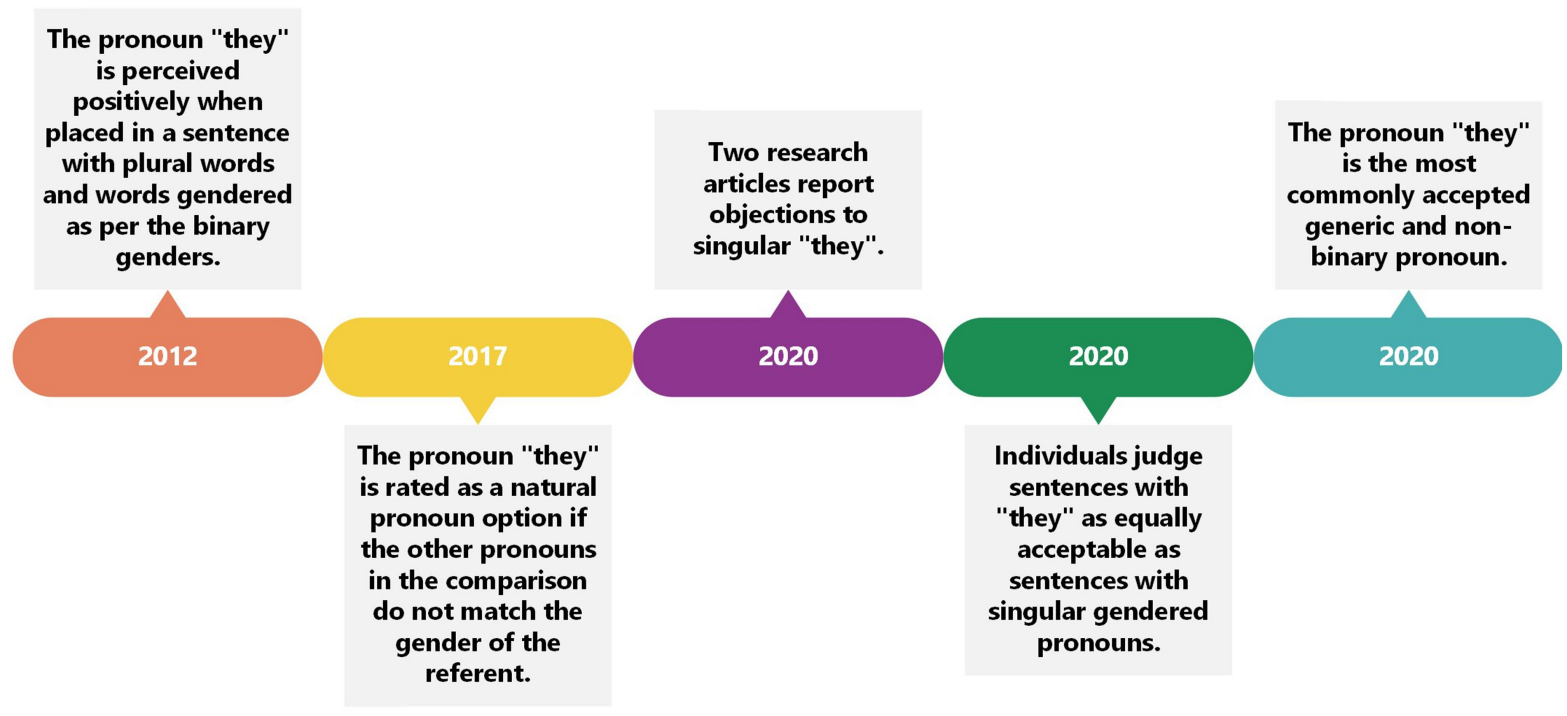
Timeline to show the acceptance of the pronoun “they” through the years.

The results reflect a shift in acceptance, although it appears that there is still a debate of prescriptivist bias in terms of acceptance. Bodine (1975) explained that from the 19th century onwards, prescriptive grammarians identified the pronoun “they” as plural only, and it appears this interpretation is still rooted in the English language today. However, the data suggests that the pronoun “they” is still accepted as a generic and as a non-binary pronoun, although its meaning and interpretation has varied in recent years. It may be that new alternative pronouns (neopronouns), such as “ey,” “em,” “xe,” and “ze,” which have no historical attachments to them, are easier to perceive and be accepted as non-binary and neutral. Lindqvist et al. (2019), who reported that the pronoun “they” possessed a masculine bias, also examined the neopronoun “ze,” and concluded that this pronoun could eliminate a masculine bias. In contrast, the pronoun “they” is not in the same category as neopronouns because of its familiar nature. When “they” is compared to gendered pronouns such as “he or she” it may appear to be more inclusive, but when compared to neopronouns, it possesses a masculine bias, whereas the neopronouns are perceived as neutral (Figure 4).

Overall, the findings support all three hypotheses: that “they” would become more gender-neutral in meaning, as well as increase in use and in acceptability over time. The hypothesized change is observed as the pronoun “they” is shown to be fluid and flexible, as well as a common choice of pronoun. The pronoun “they” may be gendered, neutral and genderless. Despite the prescriptivist bias debate, the pronoun “they” is still declared singular and plural. In essence, the pronoun “they” has become inclusive for all members of society and adaptable as needed.

At the same time, “they” might continue to evolve and follow the trajectory of “you,” a generic for second person singular and plural. Speakers often use additional morphology, such as “y’all” to differentiate when “you” refers to more than one individual as opposed to an individual. Over time, we may begin to hear phrases such as “they all” to differentiate the third person singular and plural. Future research will be able to detail this trajectory.

### Limitations

There are limitations in the current review that should be noted. Our review is based on articles that we were able to access. Although we contacted 10 researchers for unpublished research, we did not receive replies. In addition, our systematic review is confined to the articles we found in the databases we searched.

### Future directions

There are other future directions that should be addressed based on gaps in the research base. For example, research that required participants to describe a character often asked the binary (i.e., woman or man) gender of the character (Hyde, 1984; Fisk, 1985; Gastil, 1990; Bennett-Kastor, 1996) or for participants to name the character (Moulton et al., 1978; Khosroshahi, 1989; Switzer, 1990), which were then labeled per the binary gender. This method is restrictive because it does not allow for a gender outside of the binary to be considered. This limitation may have resulted because of the year the research articles were published in. In addition, it may have occurred because the focus of some of the previous studies was to understand whether the pronoun “he” was generic or possessed a masculine bias. Nevertheless, when assessing whether pronouns elicit gendered meanings, all categories of gender need to be considered, and the choices given to the participants must not be restricted to the binary. Two exceptions are Bradley et al. (2019) and Lindqvist et al. (2019), which included photographs of individuals who were non-binary (judged by reliability rating) and in the gender binary.

The population of each study must be considered as well, and future studies must consider recruiting varied samples. Hyde (1984), Fisk (1985), Switzer (1990), Bennett-Kastor (1996), and Conkright et al. (2000) were the only researchers to explore pronouns with a child sample (3–12 years-old). This omission suggests children are underrepresented in the topic of pronouns and how these are interpreted, and it cannot be established how children understand the pronoun “they.” The available literature suggests that “they” possesses masculine connotations (Hyde, 1984; Switzer, 1990) and at the same time, functions in a non-masculine biased way (Fisk, 1985), such as holding a gender-neutral association (Bennett-Kastor, 1996), when compared to “he or she” alternatives (Switzer, 1990); girls perceive the pronoun “they” as more generic than boys (Conkright et al., 2000). There is not sufficient research on the pronoun “they” with children to suggest what their interpretation of the pronoun “they” is and how children obtained this understanding. Of the remaining studies that conducted empirical research, their samples were all adults. Thus, the conclusions that emerged from the review can be applied to adults. However, the majority of adult samples were university students, and whilst an accessible sample, it may be considered a biased sample due to demand characteristics or their educational level. Future research should expand on the kind of adult sample recruited so that samples vary in age, levels of education, ethnicity, sexuality, and gender identity to obtain a representative idea of how multiple adult samples interpret the pronoun “they.”

## Conclusion

Overall, what the data suggests is that there has been a shift in terms of interpretation, usage, and acceptance of the pronoun “they.” However, given the gaps in the literature (e.g., age of sample), further research is necessary. First, future research needs to investigate neopronouns and how to integrate neopronouns in mainstream society, including their interpretation, acceptance, usage, etc. As mentioned, Sweden introduced a third person pronoun, exclusively genderless/generic: “hen.” There was an increase in positive attitudes between 2012 to 2018 (Gustafsson Sendén et al., 2015, 2021). Thus, whilst language reforms are difficult to achieve (Paterson, 2014), it is not impossible, and further research is required into neopronouns to assess whether a similar reform can be achieved in the English language.

Second, research should continue to investigate the pronoun “they” with multiple population types (i.e., children and adults) to decipher how it is interpreted (i.e., a masculine biased pronoun, a feminine biased pronoun, a generic pronoun, a non-binary pronoun, a plural pronoun). There are many benefits that come with understanding how the pronoun “they” is interpreted, and in turn, how the pronoun can be integrated into multiple aspects of society. For example, research needs to investigate if “they” can be integrated into education and weaken related gender-stereotypes. Future research could focus on the impact the pronoun “they” may have on gender-stereotypes as well as the impact the pronoun “they” has on prejudice. For example, integrating the use of “they” in everyday language may reduce levels of sexism or racism. Language is a powerful tool, and as the linguistic relativity hypothesis suggests, the languages we speak influence the way we think about the world (Whorf, 1956). This influence of language on thought has been demonstrated in various domains, including grammatical gender (e.g., Bassetti, 2007; Sato and Athanasopoulos, 2018). Thus, further investigation of the pronoun “they” is crucial. Whilst a language reform may not solve these societal impacts of bias, it may contribute to creating greater inclusivity and equality for all.

## Data availability statement

The original contributions presented in the study are included in the article/Supplementary material, further inquiries can be directed to the corresponding author.

## Author contributions

MBDC: Conceptualization, Data curation, Formal analysis, Funding acquisition, Investigation, Methodology, Project administration, Resources, Writing – original draft, Writing – review & editing, Visualization. HT: Conceptualization, Methodology, Project administration, Supervision, Validation, Writing – review & editing, Visualization. AG: Conceptualization, Methodology, Project administration, Supervision, Validation, Writing – review & editing, Visualization.
