# Supplementary material for: “What do they mean?” a systematic review on the interpretation, usage and acceptability of “they”
Source: Front Psychol. 2024 Apr 5;15:1253356. doi: 10.3389/fpsyg.2024.1253356 (PMC11026696; doi:10.3389/fpsyg.2024.1253356)
Supplement: Supplementary file 1 [file Table_1.docx]

| **Table S1.**  *Initial search terms, combinations, and results* | | | | | |
| --- | --- | --- | --- | --- | --- |
| Database | Search term combinations | | | Number of papers emerged | Number of papers selected |
| PsychINFO | *Gender-Neutral* | *They* | *Pronouns* | 14 | 3 |
|  | *Interpretation* | *They* | *Pronouns* | 95 | 2 |
|  | *Interpretation* | *Gender* | *Pronouns* | 43 | 0 |
|  | *Gender-Neutral* | *Pronouns* |  | 25 | 2 |
| **Total** |  |  |  | 177 | 7 |
|  | | | | | |
| Web of Science | *Gender-Neutral* | *They* | *Pronouns* | 32 | 2 |
|  | *Interpretation* | *They* | *Pronouns* | 19 | 0 |
|  | *Interpretation* | *Gender* | *Pronouns* | 81 | 0 |
|  | *Gender-Neutral* | *Pronouns* |  | 7 | 0 |
| **Total** |  |  |  | 139 | 2 |
