# Supplementary material for: “What do they mean?” a systematic review on the interpretation, usage and acceptability of “they”
Source: Front Psychol. 2024 Apr 5;15:1253356. doi: 10.3389/fpsyg.2024.1253356 (PMC11026696; doi:10.3389/fpsyg.2024.1253356)
Supplement: Supplementary file 2 [file Table_2.docx]

| **Table S2.**  *Revised search terms, combinations, and results* | | | | | |
| --- | --- | --- | --- | --- | --- |
| Database | Search Term Combinations | | | Number of Papers Emerged | Number of Papers Selected |
| PsychINFO | *Gender** | *Pronoun** | *Interpretation* | 71 | 0 |
|  | *Gender** | *Prenominal** | *Interpretation* | 1 | 0 |
|  | *Gender** | *Epicene Pronoun** | *Interpretation* | 0 | 0 |
|  | *Gender** | *Epicene Pronoun** |  | 4 | 0 |
|  | *Interpretation* | *Gender-Neutral Pronoun* |  | 1 | 0 |
|  | *Interpretation* | *They* | *Pronoun** | 204 | 0 |
|  | *Interpretation* | *Gender** | *Singular They* | 0 | 0 |
|  | *Interpretation* | *Singular They* |  | 3 | 0 |
|  | *Gender** | *Singular They* |  | 13 | 0 |
|  | *Gender** | *Prenominal** |  | 8 | 0 |
|  | *Grammatical Gender** | *Pronoun** |  | 44 | 1 |
|  | *Grammatical Gender** | *Epicene Pronoun** |  | 1 | 0 |
|  | *Grammatical Gender** | *Prenominal** |  | 6 | 0 |
|  | *Comprehension* | *They* | *Pronoun** | 220 | 2 |
|  | *Gender** | *Pronoun** | *Imagery* | 8 | 1 |
|  | *Gender** | *Singular They* | *Imagery* | 0 | 0 |
|  | *Interpretation* | *Singular They* | *Imagery* | 0 | 0 |
|  | *Interpretation* | *Pronoun** | *Imagery* | 5 | 0 |
|  | *Comprehension* | *Pronoun** | *Imagery* | 8 | 0 |
| **Total** |  |  |  | 597 | 4 |
|  | | | | | |
| Web of Science | *Gender** | *Pronoun** | *Interpretation* | 186 | 0 |
|  | *Gender** | *Prenominal** | *Interpretation* | 3 | 0 |
|  | *Gender** | *Epicene Pronoun** | *Interpretation* | 0 | 0 |
|  | *Gender** | *Epicene Pronoun** |  | 13 | 2 |
|  | *Interpretation* | *Gender-Neutral Pronoun* |  | 5 | 0 |
|  | *Interpretation* | *They* | *Pronoun** | 886 | 0 |
|  | *Interpretation* | *Gender** | *Singular They* | 7 | 0 |
|  | *Interpretation* | *Singular They* |  | 369 | 0 |
|  | *Gender** | *Singular They* |  | 213 | 0 |
|  | *Gender** | *Prenominal** |  | 15 | 0 |
|  | *Grammatical Gender** | *Pronoun** |  | 220 | 0 |
|  | *Grammatical Gender** | *Epicene Pronoun** |  | 4 | 0 |
|  | *Grammatical Gender** | *Prenominal** |  | 9 | 0 |
|  | *Comprehension* | *They* | *Pronoun** | 352 | 0 |
|  | *Gender** | *Pronoun** | *Imagery* | 19 | 0 |
|  | *Gender** | *Singular They* | *Imagery* | 4 | 0 |
|  | *Interpretation* | *Singular They* | *Imagery* | 0 | 0 |
|  | *Interpretation* | *Pronoun** | *Imagery* | 49 | 0 |
|  | *Comprehension* | *Pronoun** | *Imagery* | 13 | 0 |
| **Total** |  |  |  | 2367 | 2 |
